# Diagnostic and Practical Value of Abbreviated Contrast Enhanced Magnetic Resonance Imaging in Breast Cancer Diagnostics

**DOI:** 10.3390/cancers14225645

**Published:** 2022-11-17

**Authors:** Martin Drinković, Ivan Drinković, Dražen Milevčić, Filip Matijević, Vlatka Drinković, Antonio Markotić, Tade Tadić, Davor Plavec

**Affiliations:** 1 Department of radiology, Polyclinic Drinković, 10 000 Zagreb, Croatia; 2 Department of pulmonology, Srebrnjak Children’s Hospital, 10 000 Zagreb, Croatia; 3 School of Medicine, University of Mostar, 88 000 Mostar, Bosnia and Herzegovina; 4 Department of radiology, University Hospital Centre Split, 21 000 Split, Croatia; 5 School of Medicine, University of Split, 21 000 Split, Croatia; 6 Medical Faculty, University Josip Juraj Strossmayer, 31 000 Osijek, Croatia

**Keywords:** abbreviated MRI protocol, breast cancer, magnetic resonance imaging, full diagnostic protocol

## Abstract

**Simple Summary:**

Breast cancer is the most common cancer in women and requires early diagnosis and treatment. Although MRI is the most efficient method of detecting breast cancer, its standard protocol is time-consuming and expensive, making it less accessible. The aim of this study was to compare the diagnostic accuracy of the modified abbreviated MRI protocol (AMRP) to that of the standard magnetic resonance protocol. The study shows that both MRI protocols have comparable accuracy (specificity and sensitivity) for detecting breast cancer. These findings suggest that the abbreviated MRI protocol can reduce the examination and image reading time, as well as costs, while maintaining the diagnostic accuracy of a full diagnostic protocol. The key finding is that AMRP can provide appropriate diagnostic accuracy for breast cancer for a much wider population and reduce MRI waiting lists.

**Abstract:**

Background: Although MRI is the most efficient method of detecting breast cancer, its standard protocol is time-consuming and expensive. The objective of this study was to compare the diagnostic accuracy of the modified innovative abbreviated MRI protocol (AMRP) and the standard magnetic resonance protocol (SMRP) when detecting breast cancer. Methods: The research involved 477 patients referred for breast MRI due to suspected lesions. They were randomly assigned to the AMRP group (N = 232) or the SMRP group (N = 245). The AMRP comprised one native (contrast-free) and four post-contrast dynamic sequences of T1-weighted volume imaging for breast assessment (VIBRANT) and 3d MIP (maximum intensity projection) lasting for eight minutes. All the patients underwent a core biopsy of their lesions and histopathological analysis. Results: The groups were comparable regarding the pre-screening and post-diagnostic characteristics and were of average (±SD) age at breast cancer diagnosis of 53.6 ± 12.7 years. There was no significant difference between the two protocols in terms of specificity or sensitivity of breast cancer diagnosis. The sensitivity (95% Cis) of the AMRP was 99.05% (96.6–99.9%), and its specificity was 59.09% (36.4–79.3%), whereas the sensitivity of the SMRP was 98.12% (95.3–99.5%) and its specificity was 68.75% (50.0–83.9%). Most of the tumors comprised one solid lesion in one of the breasts (77.3%), followed by multicentric tumors (16%), bilateral tumors (4.3%), and multifocal tumors (1.7%). The average size of tumors was approximately 14 mm (ranging from 3 mm to 72 mm). Conclusion: Our innovative AMR protocol showed comparable specificity and sensitivity for the diagnosis of breast cancer when compared to SMRP, which is the “gold standard” for histopathological diagnosis. This can lead to great savings in terms of the time and cost of imaging and interpretation.

## 1. Introduction

Breast cancer is the most common malignant disease in women, and ~20% of cases reach an advanced stage by the time it is diagnosed [1,2,3]. According to the latest available data from the Public Health Institute of the Republic of Croatia, more than 2950 patients are diagnosed with breast cancer each year, an incidence rate of approximately 143.2 women per 100,000 inhabitants, and the European average is approximately 102.5. According to the latest available epidemiological data, breast cancer was the third most common cause of death in the female population in 2020, and the rate of breast cancer-related mortality in Croatia is among the highest in Europe. In 2020, the mortality rate was more than 32/per 100,000 inhabitants, which is a total of 752 women, whereas the European average is approximately 20/per 100,000 [4]. Delays in the diagnosis and treatment affect mortality because one of the most important prognostic factors of survival in breast cancer is the clinical stage [5,6].

The current methods of choice for verifying breast cancer are mammography (MMG) and ultrasound (US) when indicated. Final diagnosis requires a biopsy [7,8,9,10]. A method that is being used more frequently is magnetic resonance imaging (MRI) with a contrast agent [11,12,13]. This method provides a contrast image that shows the imbibition dynamics of the cancerous mass and the surrounding tissue affected by cancer [14]. MRI is the most efficient imaging method for detecting breast cancer because it is highly sensitive (85–100%), but it is somewhat less specific (55–90%) [15]. The advantage of this method is the avoidance of exposure to harmful ionizing radiation.

Depending on the manufacturer of the MRI device and the sequences used, the standard protocol for breasts lasts approximately 30 min to an hour (sagittal, axial, coronal sections), so this kind of examination is difficult to conduct in all women with the indication. This is especially the case for women who suffer from claustrophobia and cannot tolerate the examination [16,17,18]. By reviewing related publications [18,19,20,21,22] and backed by clinical practice, we have developed our own modified innovative abbreviated MRI protocol (AMRP) for breast examination with contrast, which lasts between 7 and 10 min and enables the early diagnosis of breast cancer.

The standard protocol for breast MRI (SMRP) comprises T1-weighted images before applying contrast and T1-weighted images after applying contrast to show the dynamics of the collection of contrast in the lesion because cancers larger than 2 mm show signs of neovascularization [23]. This is presented as the leakage of blood vessels inside the cancerous mass, which also leads to the quick washout of the contrast agent. In the standard protocol, T2-weighted images are also added [16,17]. The abbreviated MRI protocols are currently not standardized, but the average imaging time is less than 10 min. We believe that each examination should be reduced to a minimum duration so that patients can enjoy greater availability of MRI scans and a more pleasant examination without losing diagnostic accuracy when compared to the standard protocol used for a breast MRI.

Consequently, the hypothesis behind this research was that the modified innovative AMRP with the use of shortened sequences could improve the diagnostics of breast cancer in terms of shortening the examination time without compromising the sensitivity or specificity of the method compared to the SMRP by showing the bilaterality or multifocality of the lesion and the extent to which axillary lymph nodes are affected. To prove the research hypothesis, we defined specific objectives: (1) calculate the sensitivity and specificity of the AMRP for breast cancer according to the histopathological diagnosis; (2) compare the sensitivity and specificity with the SMRP examination conducted with the same MRI device; (3) assess the interrater agreement for the AMRP; (4) compare the time required for the AMRP vs. SMRP.

## 2. Subjects and Methods

This was a single-site randomized cross-sectional comparative diagnostic accuracy study in female subjects referred for breast cancer diagnosis conducted at Polyclinic Drinković in Zagreb over the course of six years. The work described was carried out in accordance with the Code of Ethics of the World Medical Association (Declaration of Helsinki) and was approved by the Ethics Committee of Policlinic Drinković. Informed consent was obtained from all participants.

A total of 477 female patients aged 20 to 84 were included. The inclusion criteria were either self-referral (finding the lesion on self-assessment) or a referral by a physician due to suspicious changes in breasts (MMG or US). The exclusion criteria were patients with contraindications for MRI or an allergy to a contrast agent. Patients were randomized to the AMRP or SMRP group using a predefined randomization sequence with an approximate 1:1 ratio. A total of 232 MRI scans were conducted with the AMRP, and 245 scans were completed with the SMRP. Premenopausal women were referred to imaging between the 7th and 14th day of the menstrual cycle. Of the 477 patients, 17 had a positive personal history of breast cancer.

### 2.1. MRI and Analysis

**Figure 1:**
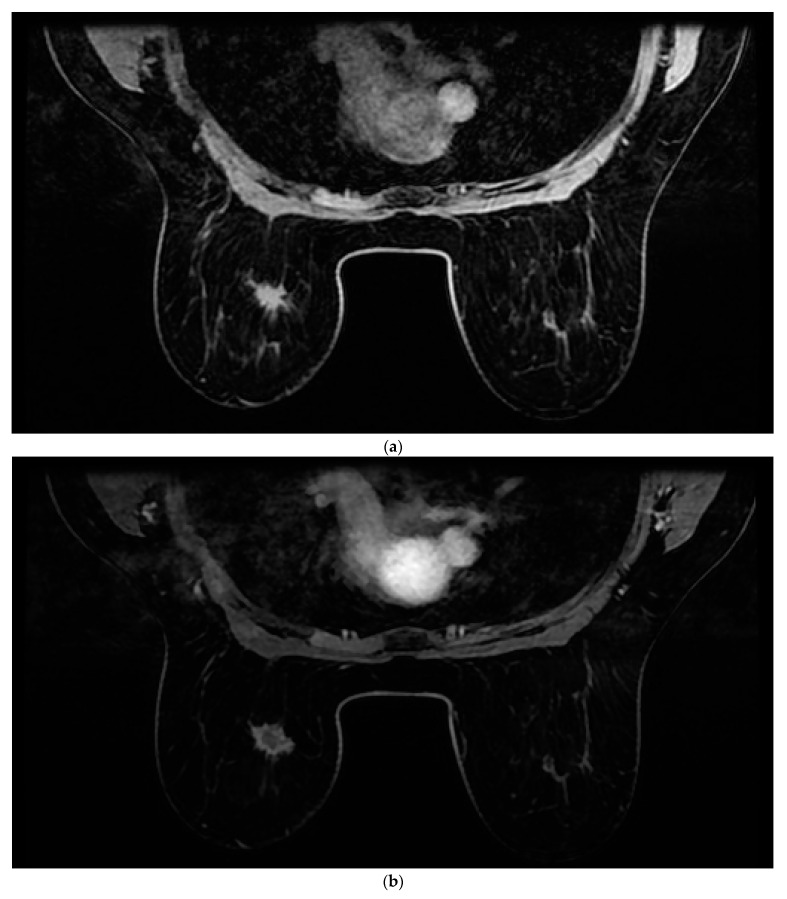

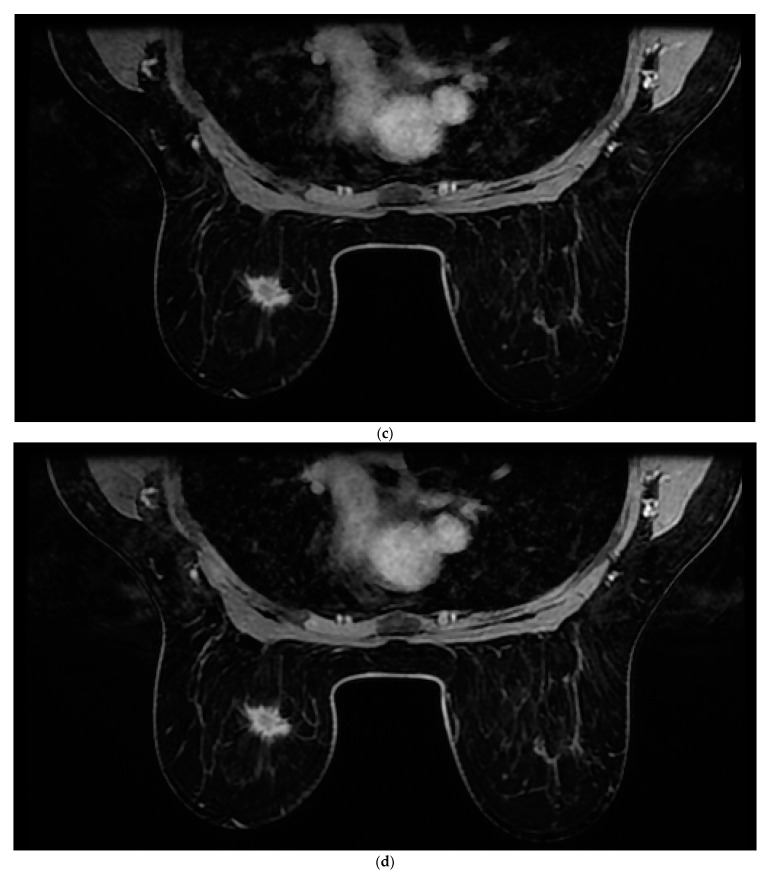

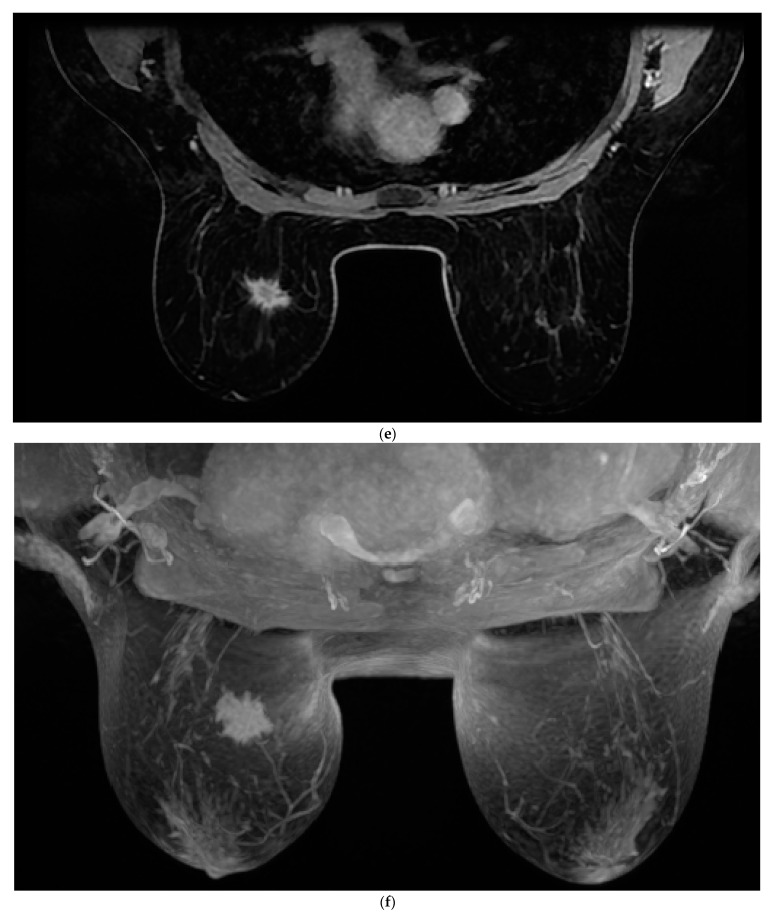
Images of a 43-year-old woman with a new 2 cm left breast mass (invasive ductal carcinoma—IDC). (**a**) Native. (**b**) First postcontrast sequence. (**c**) Second postcontrast sequence. (**d**) Third postcontrast sequence. (**e**) Fourth postcontrast sequence. (**f**) Maximum intensity projection (MIP).

All patients were assessed for breast cancer on the MR 1.5 T (Signa Explorer, GE) MRI device with an 8-channel coil modified for breast examination. Women were placed in the prone position with both breasts positioned asymmetrically in the coil. The contrast agent used was gadolinium at a dose of 0.2 mL/kg + (Gadovist Bayer Pharma AG, Berlin, Germany). The AMRP comprised one native and four post-contrast images obtained through dynamic T1-weighted volume imaging breast assessment (VIBRANT) sequences and a 3d maximum intensity projection (MIP) lasting between 6 and 9 min. The image sequences used are shown in Figure 1. Rapid contrast uptake of a lesion with washout is characteristic of invasive carcinomas and therefore differs from benign lesions that imbibe more slowly or do not wash out. For the SMRP, pre-contrast MRI sequences were used, which include turbo spin echo (TSE) T1 images (T1WI), transversal TSE T2 images (T2WI) with fat suppression, sagittal TSE T2WI with fat suppression, T1-weighted volume imaging for breast assessment (VIBRANT) images, and 3d MIP where the same contrast agent was used, all lasting between 25 and 35 min. All the images were interpreted by two radiologists independently of each other (one of whom had more than 25 years of experience in reading MRIs, and the other who had 5 years of experience). Each radiologist reached a conclusion based on Z-1 (AMRP and 3d MIP). The diagnosis was made based on a US categorization system called BI-RADS (Breast Imaging Reporting and Data System). Each radiologist reached their conclusion based on Z-2 (SMRP and 3d MIP) and Z-1. All MRI results were categorized based on BI-RADS. Categories 1, 2, or 3, according to the BI-RADS classification, were considered negative, and categories 4 or 5 were considered positive for breast cancer.

### 2.2. Histopathological Verification

All patients underwent a core needle biopsy (CNB) of the detected lesions. Biopsy was performed using the BARD^®^ MAGNUM^®^ Reusable Core Biopsy System, with additional control by ultrasound or MRI. The biopsy specimens were sent for histopathological processing, where the following parameters were examined: histological sub-type of tumor, tumor size, estrogen receptor, progesterone receptor, and HER2 status. The size of the tumor was defined by the imaging method. In multifocal breast cancer, the sum of the diameters of the malignant lesions was calculated.

### 2.3. Statistical Analysis

Histopathological results were used as a reference standard for diagnosis. Categorical variables were presented as numbers (N) and percentages (%) and compared between groups with the chi-square test or Fisher’s exact test when appropriate. Continuous variables are presented as median with interquartile range (IQR) or mean ± standard deviation (SD) and were compared using the Mann–Whitney U test or Student’s *t*-test. Cohen’s kappa test was used to assess agreement between rates. ROC analysis was performed, and sensitivity, specificity, positive and negative likelihood ratios (PLR and NLR), positive and negative predictive values (PPV and NPV), and area under the curve (AUC) were calculated and presented with 95% confidence intervals (95% CIs). *p* < 0.05 was considered statistically significant for all analyses. The statistical analyses were performed, and the figures were created in GraphPad Prism, version 6 (GraphPad Software Inc, La Jolla, CA, USA) and MedCalc Statistical Software, version 14.8.1 (MedCalc Software, Ostend, Belgium).

## 3. Results

A total of 477 patients with suspected breast cancer were recruited for MRI and completed the diagnostic procedure. Of these, 232 underwent imaging with AMRP, and 245 underwent imaging with SMRP. The basic characteristics of the subjects and their characteristics according to the MRI protocol are presented in Table 1.

The average age of patients with breast cancer diagnosis was 53.6 ± 12.7 years. The histopathology confirmed 423 cases of cancer and 54 benign lesions. Most of the tumors were a single solid lesion in one breast (77.3%), followed by multicentric tumors (16%), bilateral tumors (4.3%), and multifocal tumors (1.7%). The histological types of the cancers by frequency were as follows: 319 (75.6%) invasive ductal carcinomas (IDC), 58 (13.7%) ductal carcinomas in situ (DCIS), 15 (4.2%) mixed IDC and DCIS, 16 (3.5%) invasive lobular carcinomas, 9 (2.5%) lobular carcinomas in situ, 6 (1.6%) pathologically unclear, 5 (1.5%) papillary carcinomas, 4 (1.0%) Paget’s diseases, and 2 (0.4%) phyllodes tumors. When the histopathological analysis was negative for cancer, the lesions were mostly cases of adenosis and fibroma. According to our research, the first menstrual cycle before the age of 12 was not found to be a risk factor for breast cancer, but there were significantly more breast cancer cases in women whose menopause began before the age of 55 than in those whose menopause came later. The average tumor size was approximately 14 mm (ranging from 3 mm to 72 mm).

In the AMRP group, 210 cases were verified to be cancers. The AMRP MRI categorized 208 as cancers. Nine lesions were characterized as suspicious, but the histopathology verified them as benign lesions. Thirteen lesions were characterized as benign but were still referred to as fine needle aspiration due to other medical findings that suggested otherwise (MMG or US).

In the SMRP group, 213 cases were verified as cancers. The SMRP MRI categorized 209 as cancers. Ten lesions were characterized as cancers, but the histopathology verified them as benign lesions. Twenty-two lesions were characterized as benign but were still referred to as fine needle aspiration due to other medical findings that suggested otherwise (MMG or US).

When we compared the interrater agreement using the kappa test, we found a moderate to fair interrater agreement between the two raters (kappa = 0.56, 95% CI 0.46 to 0.66).

The sensitivity of the AMRP was 99.05% (96.6–99.9), and its specificity was 59.09% (36.4–79.3), whereas the sensitivity of the SMRP was 98.12% (95.3–99.5), and its specificity was 68.75% (50.0–83.9). PPV for the AMRP was 95.9%, and NPV was 86.7%, whereas PPV for the SMRP was 95.4% and NPV was 84.6% (Table 2).

**Figure 2:**
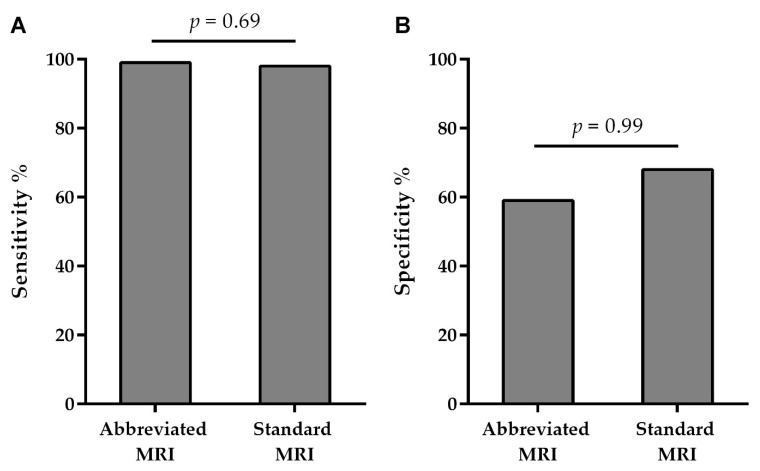
Comparison of the sensitivity (**A**) and specificity (**B**) of the two protocols used (abbreviated vs. standard MRI protocol).

We found no differences between the AMRP and the SMRP when comparing the specificity and sensitivity of the MRI examination for breast cancer diagnosis (Table 2 and Figure 2).

The diagnostic accuracy of both the AMRP and SMRP was not significantly different between raters (*p* = 0.080 and *p* = 0.250, respectively, ROC analysis) or between methods for the first or the second rater (*p* = 0.167 and *p* = 0.391, respectively, ROC analysis).

## 4. Discussion

We have shown that both protocols (the AMRP and SMRP) present comparable results regarding the specificity and sensitivity of the MRI examination for breast cancer. The groups used for the comparison were large enough and had comparable characteristics (age, tumor type, cancer type, etc.). In this respect, our results are in line with the results of previous studies comparing standard and other abbreviated modified protocols for breast cancer detection. We have also shown an improvement in the sense that the AMRP lasted only 8 min on average (depending on the size of the breast, between 6 and 9 min). The SMPR, on the other hand, required 25–35 min. The reading time for the MIP sequences was approximately 3 s per sequence (ranging between 2 and 8 s), with an average time for the entire AMRP of 2 min (ranging between 1.5 and 3 min), whereas the SMRP took approximately 5 min. Unlike our AMRP, other researchers analyzed abbreviated protocols that mainly comprised a T2 image, a STIR sequence, or one or more post-contrast dynamic sequences. Kuhl et al. [18] significantly reduced the examination and image reading time while preserving the diagnostic accuracy of a full diagnostic protocol. This was achieved in the context of a screening performed on 443 women using an MRI protocol consisting of an unenhanced T1-weighted and first contrast-enhanced T1-weighted sequence, subtraction imaging, and a single MIP image, lasting a total of 3 min. This was further elaborated on by Mango et al. [24] with an abbreviated protocol consisting of fat-saturated T1-weighted pre-contrast, early post-contrast T1, and subtraction MIP sequences, with the imaging protocol being performed in approximately 10–15 min. Other studies that provide evidential support for this practice are Grimm et al. [25], Harvey et al. [26], and many others after them. Based on their knowledge and experiences, we reached the conclusion that T2 or STIR sequences will not be of use to us if we keep in mind the fact that tumors larger than 2 mm have increased angiogenesis and consequently increased imbibition, which makes them wash out faster. T2 weighted sequences were of no use to us either because they are mostly used to diagnose benign changes. Similarly, we cannot rely on one or more postcontrast sequences because they are not sufficient enough to show us the lesion imbibition dynamics. Benign lesions normally present with initially slow or delayed imbibition, whereas quick initial imbibition and quick washout are highly indicative of a malignant mass. Therefore, we believe that one or two postcontrast sequences would not be sufficient for adequate analysis and would lead to many repeated examinations, which would only increase costs and provide no benefits.

Considering that the time spent on diagnosis is crucial to the outcome, the possibility of using the same resources better and ensuring their faster and greater availability, thus allowing for early diagnosis, is essential for breast cancer. The average waiting time for examinations in hospitals in our country is >3 months (in category 1 national hospitals 133 days, in category 2 hospitals 167 days, and in category 3 hospitals 106 days [27]). However, the exact number of patients on the waiting list is not available. The AMRP requires much less time compared to the SMRP (9 (7–10) min compared to 28 (25–35) min), which means that during one shift, it would be possible to examine 46 patients using the AMRP compared to 15 patients when using the SMRP. This would mean that we could screen approximately 30 patients more each day. This would make it possible to shorten the current waiting lists down to one-third of their current level or even more in the long run.

The price of an MRI protocol for breast examination based on the prices of the Croatian Health Insurance Fund in 2022 was HRK 1016 (HRK 716 + 300 for the contrast agent, which is EUR ~135), whereas in private institutions, it ranges from HRK 1600 to 2200, i.e., EUR 213 to 293. By shortening the protocol, one could also reduce its price, which could ultimately result in financial savings as well.

Based on the literature, it is evident that breast cancer screening via the standard MRI protocol also has financial benefits in patients of a certain age, patients with genetic predispositions, and women with dense glandular parenchyma.

Geuzinge A. et al. found that using the model of MRI screening every 18 months for the ages 35–60, with a subsequent national screening program until 75 years, provides an efficient and cost-effective strategy, with an incremental cost-effectiveness ratio (ICER) just below the threshold of GBP 20,000. [28]. Based on numerous studies, MRI breast cancer screening provides enough evidence to suggest it is a much better screening method than MMG. This is especially the case in women with a family history of breast cancer and genetic predisposition, allowing the detection of cancers at an earlier stage, thus reducing the use of adjuvant chemotherapy and decreasing mortality [29]. Bakker et al. found that “MRI screening in women with extremely dense breast tissue and normal results on MMG resulted in the diagnosis of significantly fewer interval cancers than MMG alone during a 2-year screening period” [30,31]. 

Given that an appropriate network of available MRI machines must be provided to perform MRI screening, the introduction of the AMRP would enable three times more availability than the SMRP, additionally reducing costs.

We have proven the hypothesis of this paper, namely that breast cancer diagnostics via MRI can be improved by shortening the duration of the examination in comparison to the conventional MRI protocol without losing the diagnostic accuracy of the method.

The limitations of this study lie in the fact that the research was conducted in a single private polyclinic where the examination is not reimbursed by the Croatian Health Insurance Fund. Consequently, this could introduce certain biases in terms of subject selection. Our subjects were probably of higher socioeconomic status, so they may have been more aware of the necessity for a comprehensive examination and for earlier and regular visits to their physician for the purposes of undergoing preventive examinations. In addition, MRIs were more easily accessible to them. Additionally, the same patients were not examined using both protocols, but the groups were big enough and were shown to be comparable. The strength of this study is that all patients underwent histopathological testing as a reference for the diagnostic accuracy assessment of both protocols.

## 5. Conclusions

Our modified innovative abbreviated MRI protocol (AMRP) showed fully comparable results in terms of sensitivity and specificity for breast cancer diagnosis when compared to the standard MRI protocol. When used for the detection of breast cancer, the AMRP significantly shortened the duration of both the examination and interpretation phases and increased MRI patient availability. Thus, the AMRP has the potential to provide the earliest possible diagnosis of breast cancer to a wide population of patients at risk.

## Figures and Tables

**Table 1:** Characteristics of patients referred for breast MRI according to MRI protocol (N = 477).

Variable	All (N = 477)	Abbreviated MRI (N = 232)	Standard MRI (N = 245)	*p*-Value
Age in years; mean ± SD	53.6 ± 12.7	55.0 ± 12.5	52.2 ± 12.7	0.021 ^a^
History of breast cancer				0.17 ^b^
Negative	215 (59.7)	100 (55.9)	115 (63.5)	
Positive in the family	112 (31.1)	64 (35.8)	48 (26.5)	
Positive personally	33 (9.2)	15 (8.4)	18 (9.9)	
First menstrual cycle				0.10 ^b^
Before 12 years of age	89 (25.9)	51 (29.8)	38 (22.1)	
After 12 years of age	254 (74.1)	120 (70.2)	134 (77.9)	
Menopause				0.99 ^b^
No	150 (43.4)	74 (43.3)	76 (43.4)	
Yes—before 55 years of age	158 (45.7)	78 (45.6)	80 (45.7)	
Yes—after 55 years of age	38 (11.0)	19 (11.1)	19 (10.9)	
Pregnancy				0.22 ^b^
No	57 (21.7)	32 (25.2)	25 (18.4)	
Yes—before 30 years of age	74 (28.1)	38 (29.9)	36 (26.5)	
Yes—after 30 years of age	132 (50.2)	57 (44.9)	75 (55.1)	
Number of births				0.017 ^b^
0	70 (20.1)	40 (23.1)	30 (17.0)	
1–2	252 (72.2)	114 (65.9)	138 (78.4)	
3+	27 (7.7)	19 (11.0)	8 (4.5)	
Breastfeeding				0.18 ^b^
No	101 (28.9)	57 (32.9)	44 (25.0)	
Less than 1 year	179 (51.3)	80 (46.2)	99 (56.2)	
More than 1 year	69 (19.8)	36 (20.8)	32 (18.2)	
Silicone implants				0.22 ^c^
No	346 (98.6)	171 (97.7)	175 (99.4)	
Yes	5 (1.4)	4 (2.3)	1 (0.6)	
Hormone therapy				0.30 ^b^
No	315 (91.6)	153 (90.0)	162 (93.1)	
Yes	29 (8.4)	17 (10.0)	12 (6.9)	
Hormone replacement therapy				0.50 ^c^
No	332 (97.6)	163 (97.0)	169 (98.3)	
Yes	8 (2.4)	5 (3.0)	3 (1.7)	
Chemotherapy				0.52 ^b^
No	337 (96.6)	165 (95.9)	172 (97.2)	
Yes	12 (3.4)	7 (4.1)	5 (2.8)	
Radiation				0.26 ^b^
No	336 (96.0)	164 (94.8)	172 (97.2)	
Yes	14 (4.0)	9 (5.2)	5 (2.8)	
Bilateral cancer				0.057 ^b^
No	341 (81.6)	176 (83.4)	165 (79.7)	
Yes	18 (4.3)	12 (5.7)	6 (2.9)	
Multicentric	23 (5.5)	9 (4.3)	14 (6.8)	
Multifocal	36 (8.6)	14 (6.6)	22 (10.6)	
Type of tumor				
Invasive carcinoma				
Ductal	339 (80.0)			
Lobular	15 (3.5)			
Mixed (IDC and DCIS)	9 (2.0)			
Unclear type	5 (1.4)			
Paget’s disease	4 (0.9)			
In situ carcinoma				
Ductal	41 (9.6)			
Lobular	5 (1.4)			
Miscellaneous				
NHL	3 (0.7)			
Angiosarcoma	2 (0.4)			
Tumors size in mm; median (IQR)	14.0 (10.0–20.0)	13.5 (10.0–20.0)	14.0 (10.0–20.0)	0.46 ^d^

IQR—interquartile range; SD—standard deviation; ^a^ Student’s *t*-test; ^b^ χ^2^ test; ^c^ Fisher’s exact test; ^d^ Mann–Whitney test.

**Table 2:** Diagnostic properties of the abbreviated MRI (AMRP) and standard MRI (SMRP) protocols.

	AMRP		SMRP	
	Value (95% CI)	*p*	Value (95% CI)	*p*
Sensitivity	99.05% (96.6–99.9)		98.12% (95.3–99.5)	
Specificity	59.09% (36.4–79.3)		68.75% (50.0–83.9)	
Youden index	0.58		0.67	
PLR	2.42 (1.5–4.0)		3.14 (1.9–5.3)	
NLR	0.02 (0.01–0.07)		0.03 (0.01–0.07)	
PPV	95.9% (92.3–98.1)		95.4% (91.8–97.8)	
NPV	86.7% (59.5–98.3)		84.6% (65.1–95.6)	
AUC	0.79 (0.73–0.84)	<0.001	0.83 (0.78–0.88)	<0.001

AUC—area under the curve; CI—confidence interval; NLR—negative likelihood ratio; NPV—negative predictive value; PLR—positive likelihood ratio; PPV—positive predictive value; p-value for the AUC curve.

## Data Availability

All the data generated or analyzed during this study are included in this published article. The datasets generated and/or analyzed during the current study are not publicly available due to ethics approval permission restrictions but are available from the corresponding author upon reasonable request.
